# Fibroblast origin shapes tissue homeostasis, epidermal differentiation, and drug uptake

**DOI:** 10.1038/s41598-019-39770-6

**Published:** 2019-02-27

**Authors:** Christian Hausmann, Christian Zoschke, Christopher Wolff, Maxim E. Darvin, Michaela Sochorová, Andrej Kováčik, Barbara Wanjiku, Fabian Schumacher, Julia Tigges, Burkhard Kleuser, Jürgen Lademann, Ellen Fritsche, Kateřina Vávrová, Nan Ma, Monika Schäfer-Korting

**Affiliations:** 1 Institute of Pharmacy (Pharmacology & Toxicology), Freie Universität Berlin, Königin-Luise-Str. 2 + 4, 14195 Berlin, Germany; 2 Charité - Universitätsmedizin Berlin, Charitéplatz 1, 10117 Berlin, Germany; 3 Faculty of Pharmacy in Hradec Králové, Charles University, Akademika Heyrovského 1203, 50005 Hradec Králové, Czech Republic; 4 Institute of Nutritional Science, Department of Nutritional Toxicology, University of Potsdam, Arthur-Scheunert-Allee 114-116, 14558 Nuthetal, Germany; 5 Department of Molecular Biology, University of Duisburg-Essen, Hufelandstr. 55, 45122 Essen, Germany; 6 IUF - Leibniz Research Institute for Environmental Medicine, Auf’m Hennekamp 50, 40225 Düsseldorf, Germany; 7 Institute of Biomaterial Science, Helmholtz-Zentrum Geesthacht, Kantstraße 55, 14513 Teltow, Germany

## Abstract

Preclinical studies frequently lack predictive value for human conditions. Human cell-based disease models that reflect patient heterogeneity may reduce the high failure rates of preclinical research. Herein, we investigated the impact of primary cell age and body region on skin homeostasis, epidermal differentiation, and drug uptake. Fibroblasts derived from the breast skin of female 20- to 30-year-olds or 60- to 70-year-olds and fibroblasts from juvenile foreskin (<10 years old) were compared in cell monolayers and in reconstructed human skin (RHS). RHS containing aged fibroblasts differed from its juvenile and adult counterparts, especially in terms of the dermal extracellular matrix composition and interleukin-6 levels. The site from which the fibroblasts were derived appeared to alter fibroblast-keratinocyte crosstalk by affecting, among other things, the levels of granulocyte-macrophage colony-stimulating factor. Consequently, the epidermal expression of filaggrin and e-cadherin was increased in RHS containing breast skin fibroblasts, as were lipid levels in the *stratum corneum*. In conclusion, the region of the body from which fibroblasts are derived appears to affect the epidermal differentiation of RHS, while the age of the fibroblast donors determines the expression of proteins involved in wound healing. Emulating patient heterogeneity in preclinical studies might improve the treatment of age-related skin conditions.

## Introduction

Ageing impairs cellular function and tissue regeneration, promotes disease, and ultimately leads to death. Changes in organ function occur throughout an individual’s whole lifespan, and notable differences can be seen among juvenile, adult and aged people^1^. However, the impact of ageing is underrepresented in preclinical research, even though it might contribute to the high attrition rate (90%) in current drug development^2^. Human cell-based disease models close the species gap, but the heterogeneity seen among human patients has not yet been adequately reflected^3^.

Reconstructed human skin models are used in the toxicological assessment of chemicals, and various skin disease models have been established^4^. Although skin cancer, irritant contact dermatitis, and impaired wound healing prevail in aged people, skin ageing cannot be emulated adequately *in vitro*^5^. Despite its frequent use in ageing research, artificial senescence faces major limitations as a surrogate for physiological ageing. First, senescence – a state of permanent growth arrest that cells can achieve following critical DNA damage – represents only one out of nine contributors to physiological ageing *in vivo*^1^. Second, senescence induction by artificial means over-represents the impact of senescence in aged skin. The methods that are employed achieve up to 90% senescent fibroblasts compared to the approximately 5% seen in real, aged skin^6^. Third, major differences have been described between the senescence-associated secretory phenotype and the skin ageing-associated secretory phenotype of fibroblasts^7^, a factor that might affect stromal-epithelial signalling^8^. Both toxicological test guidelines and protocols for skin disease models avoid the use of senescent or aged cells and recommend juvenile donors for the isolation of cells to reconstruct human skin^9,10^. The relevance of skin age and body region to skin homeostasis remain poorly understood. Subsequently, the predictivity of reconstructed human skin (RHS), formed of skin cells of a given age and from a given region of the body, holds in pharmacological tests remains unknown. Closing the knowledge gap is essential, especially as elderly patients in particular profit from local or transdermal drug application.

Local drug administration increases the drug concentration at the target site with potentially lower systemic adverse effects to which the elderly are more prone^11^. Transdermal drug administration makes complex and long-term treatment regimens easy to adhere to^12–14^; nevertheless, even healthy, aged skin physiology appears to affect transdermal drug administration, as shown by the reduced absorption of fentanyl in aged skin^15^.

Given that fibroblasts derived from skin cancer and atopic dermatitis determine the fate of their respective diseases^16,17^ and that fibroblasts decisively impact epidermal regeneration^18^, we hypothesized that fibroblast donor age and body region could also play central roles in tissue homeostasis, epidermal differentiation, and drug uptake. Herein, we investigate the impact of fibroblast donor age and body region on the cutaneous morphology, signalling, skin barrier function, and tissue homeostasis of RHS. We compare fibroblasts from breast skin taken from adult and aged donors as well as fibroblasts derived from juvenile foreskin. The results were attributed to donor age, donor sex, and the region of the body from which the fibroblasts were derived.

## Results

**Figure 1:**
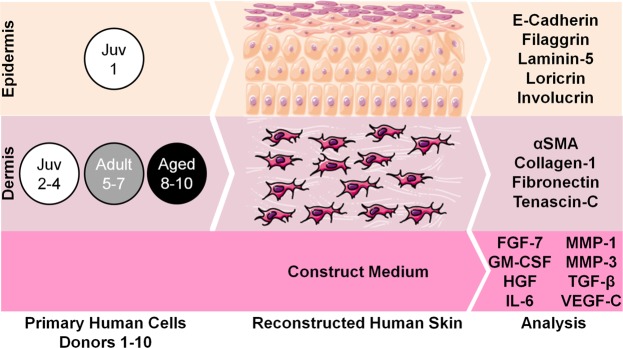
Scheme of the reconstructed human skin (RHS) used in this study. The epidermis of all RHS contained juvenile normal human keratinocytes (donor 1). Either juvenile (donor 2–4), adult (donor 5–7) or aged (donor 8–10) normal human dermal fibroblasts were used for the dermal compartment of the RHS (n = 3 for each test group). Proteins and genes were analysed from the epidermis, dermis, and medium samples. Alpha smooth muscle actin, αSMA; fibroblast growth factor-7, FGF-7; granulocyte-macrophage colony-stimulating factor, GM-CSF; hepatocyte growth factor, HGF; interleukin-6, IL-6; matrixmetalloproteinase-1/3, MMP-1/3; transforming growth factor-β, TGF-β; vascular endothelial growth factor-C, VEGF-C. This image was prepared by CH using images from Servier Medical Art under Creative Commons licence 3.0 (https://creativecommons.org/licenses/by/3.0/).

**Figure 2:**
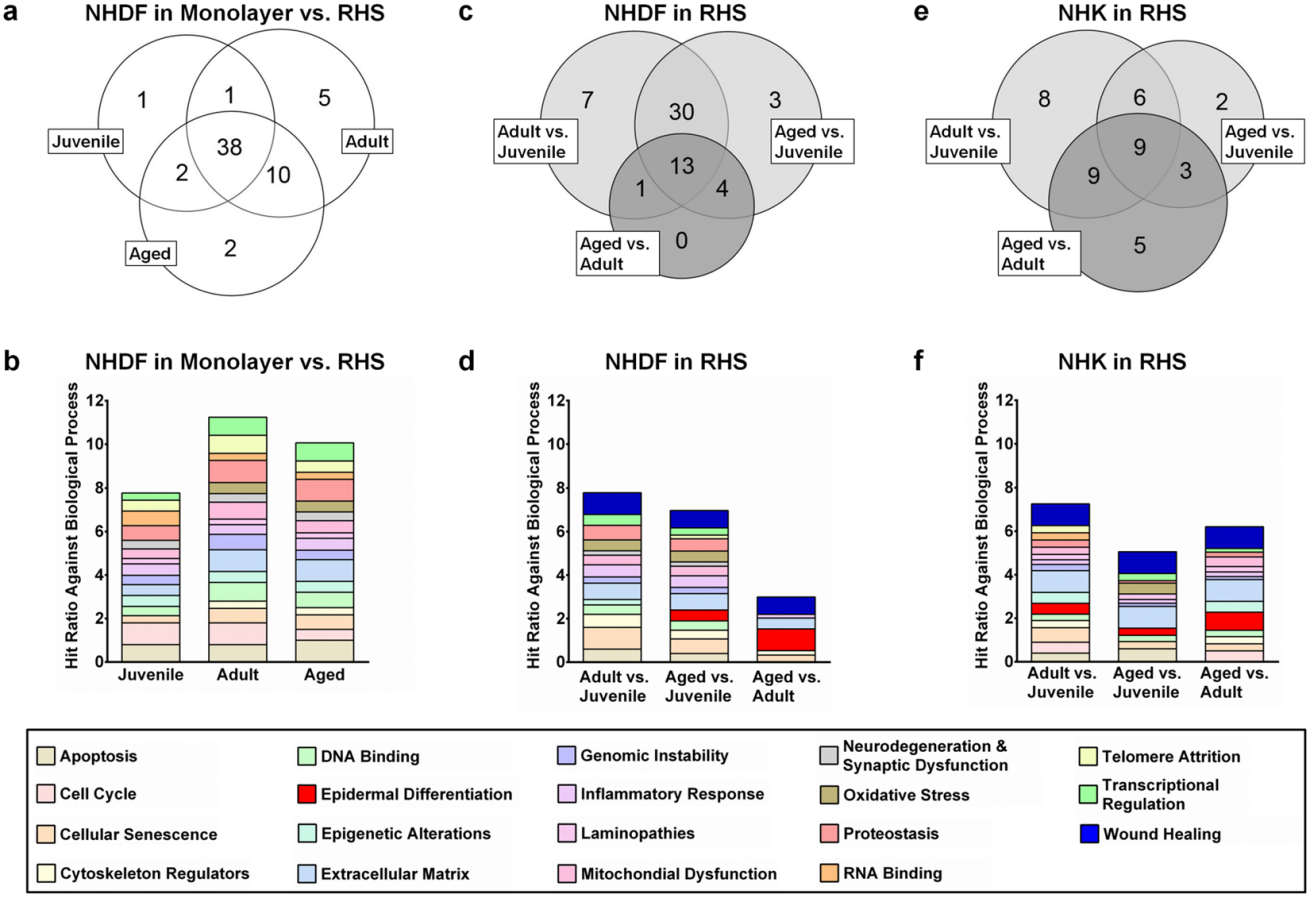
Impact of normal human dermal fibroblast (NHDF) donor age, body region, and culture on gene expression. (**a**,**c**,**e**) Venn diagrams showing the number of genes altered due to (**a**) culture conditions, (**c**,**e**) donor age (dark grey), and body region of the NHDFs (light grey). (**b**,**d**,**f**) Hit ratios of the altered genes for different biological processes. (**a**,**b**) Comparison of gene expression in NHDF monolayers and reconstructed human skin (RHS). (**c**,**d**) Comparison of gene expression in NHDFs from the RHS dermis and (**e**,**f**) in normal human keratinocytes from the RHS epidermis. The diagrams consider fold changes in gene expression > |1.3| and Ct values ≤ 35 for the 19 groups of biological processes; the maximum proportion of altered gene expression per biological process (hit ratio) = 1 (for details of biological processes see Table S1).

**Figure 3:**
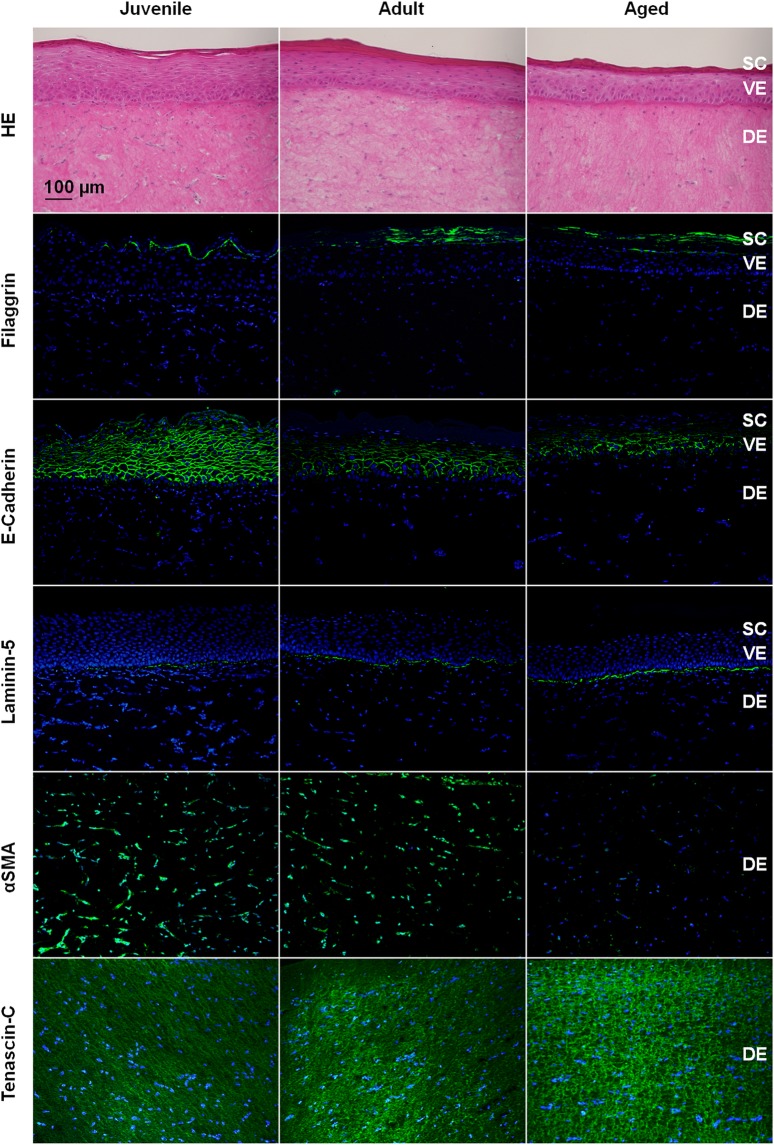
Impact of normal human dermal fibroblast donor age and body region on morphology and protein expression in reconstructed human skin. Haematoxylin and eosin staining as well as immunolocalization of filaggrin, e-cadherin, laminin-5, alpha smooth muscle actin (αSMA), and tenascin-c. *Stratum corneum*, SC; viable epidermis, VE; dermis, DE. Pictures are representative of at least three batches; scale bar = 100 µm.

**Figure 4:**
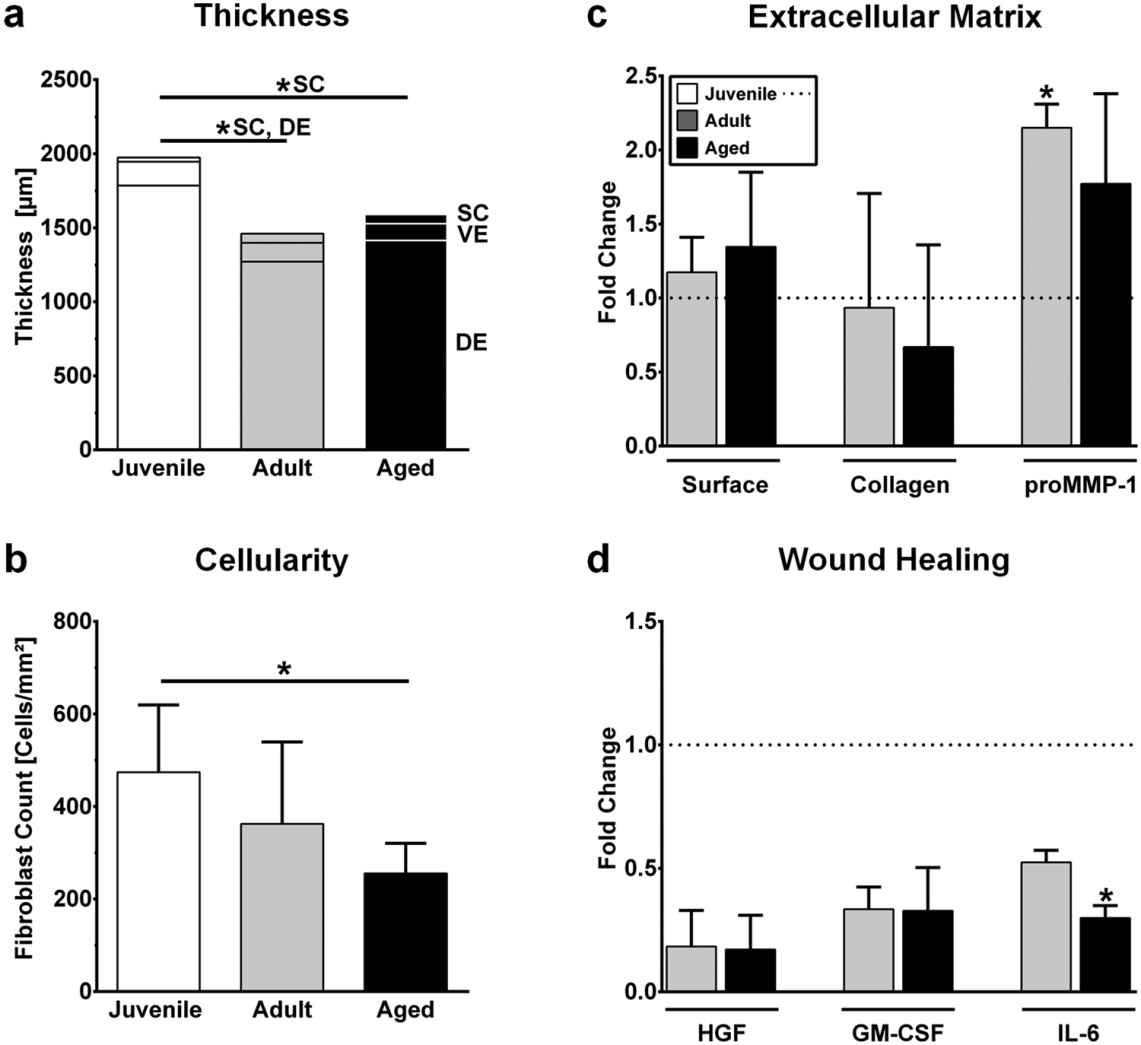
Impact of normal human dermal fibroblast (NHDF) donor age and body region on morphology and protein expression in reconstructed human skin (RHS). (**a**) Thickness of the RHS layers (*stratum corneum*, SC; viable epidermis, VE; dermis, DE). (**b**) NHDF counts. (**c**) Fold change in the surface area and collagen I content of RHS as well as the pro-matrixmetalloproteinase-1 (proMMP-1) concentration in the construct medium. (**d**) Fold change in the concentration of hepatocyte growth factor (HGF), granulocyte-macrophage colony-stimulating factor (GM-CSF), and interleukin-6 (IL-6) in the construct medium. Data are representative of at least three batches. Data are presented as the mean ± SD; *p ≤ 0.05 compared to juvenile RHS; dotted line, fold changes in comparison to juvenile RHS.

**Figure 5:**
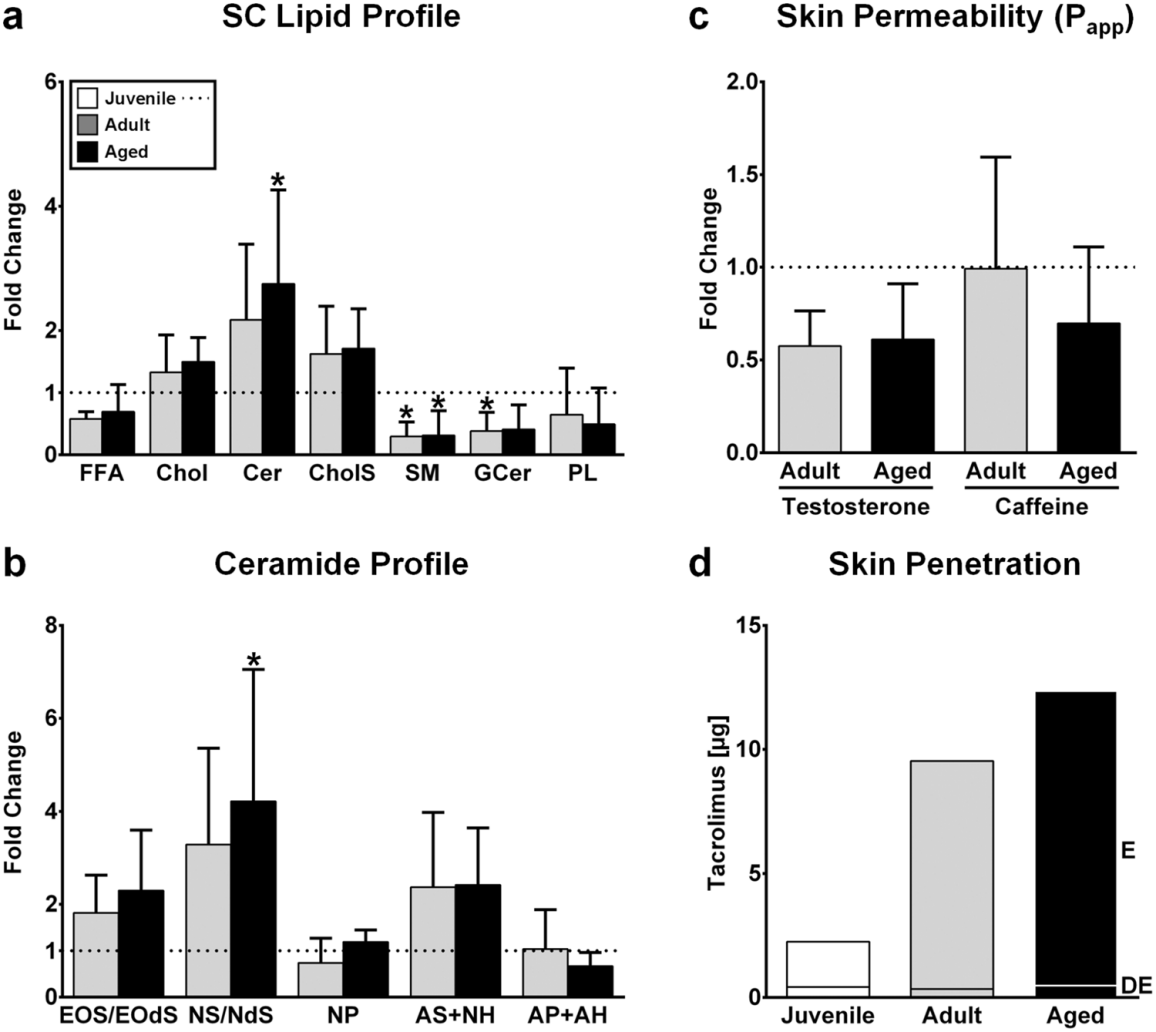
Impact of normal human dermal fibroblast donor age and body region on the *stratum corneum* (SC) lipid composition and skin barrier function and comparison to juvenile reconstructed human skin (RHS) (**a**–**c**; dotted line). (**a**) Fold change in the SC lipid composition of RHS for free fatty acids (FFA), cholesterol (Chol), ceramides (Cer), cholesterol sulfate (CholS), sphingomyelin (SM), glucosylceramides (GCer), and phospholipids (PL). (**b**) Fold change in the ceramide classes. See the Supporting Information for ceramide nomenclature. (**c**) Mean apparent permeability coefficient (P_app_) of testosterone and caffeine. (**d**) Penetration of tacrolimus from ointment into RHS. Epidermis, E; dermis, DE. Graphs depict data from three batches (**a**–**c**) or one batch (**d**) and are presented as the mean ± SD; *p ≤ 0.05 compared to juvenile RHS.

Normal human dermal fibroblasts (NHDF) from the breast skin of women aged 20–30 years (“adult”) or 60–70 years (“aged”), as well as NHDFs from the foreskin of boys <10 years old (“juvenile”), were cultured in monolayers or within the dermal compartment of RHS (Fig. 1). The gene expression profiles of the NHDFs and NHKs were analysed separately to dissect the crosstalk between both cell types (Fig. 2, Tables S3 and 4). Protein expression was investigated using tissue slices or RHS culture media (Figs 3 and 4). Finally, we studied the *stratum corneum* lipid composition and drug absorption (Fig. 5).

### The cell culture environment determines gene expression in NHDFs

The gene expression analysis initially compared juvenile, adult, and aged NHDF monolayers to their corresponding NHDFs following full culture durations in RHS (21 days of culture). Of the 59 altered gene expression levels found between the RHS and the monolayers, 38 were shared among all groups: juvenile, adult, and aged (Fig. 2a centre, Table S3). This finding suggests that most alterations occurred as a result of the fibroblasts’ cellular environment – RHS vs. monolayer – rather than as a result of their donors’ age or body region. The NHDF monolayers showed particular alterations in the expression of apoptotic (e.g., programmed cell death protein 6), proteostasis (e.g., forkhead box protein O1), and extracellular matrix (e.g., collagen type I alpha 1; Fig. 2b) genes. Interestingly, the expression levels of caspase-1 and CD14 were upregulated in juvenile NHDFs but downregulated in adult and aged cells compared to the RHS cultures. Oppositely, clusterin expression was downregulated in juvenile NHDF monolayers but upregulated in adult and aged NHDF monolayers compared to the NHDFs in RHS (Table S3).

### Body region and donor age affect gene expression

Next, we compared the gene expression profiles of juvenile, adult, and aged NHDFs following full culture durations in RHS (Fig. 2c,d). The body region of the NHDFs strongly influenced their gene expression. The regulation of 40 genes was altered between RHS containing breast skin NHDFs and RHS containing foreskin NHDFs (Fig. 2c; light grey). In comparison, the regulation of 18 genes was altered between RHS containing adult and aged NHDFs (dark grey). We observed differences in the expression of genes related to cellular senescence (e.g., caspase-1), the extracellular matrix (e.g., collagen type I alpha 1), wound healing (e.g., granulocyte-macrophage colony-stimulating factor, GM-CSF), and cell growth (e.g., hepatocyte growth factor; HGF) in adult and aged RHS compared to juvenile RHS. In fact, most genes related to wound healing clearly showed age-related differences. For example, the expression of fibronectin-1, matrix metalloproteinase-1, and tenascin-C (TNC) in RHS could be ranked aged > adult > juvenile. Juvenile RHS showed the highest expression levels of the tissue inhibitors metalloproteinase-1 and collagen type I alpha 1 (Table S4).

### NHDFs influence the gene expression profile of NHKs in RHS

Since NHDFs secrete proteins affecting NHK proliferation and differentiation, we next investigated the expression of genes related to this paracrine signalling in the epidermal compartment of RHS. The expression levels of the fibroblast growth factor-7 and transforming growth factor-beta (TGF-β) genes were investigated as they are the most relevant factors in the NHDF regulation of NHK function^19^ (Fig. 2d, grouped as “epidermal differentiation”). Their expression levels were increased from juvenile to adult RHS, and they were further increased from adult to aged RHS. More changes in gene expression were observed among the dermal layers of the different RHS than in the epidermal layers (in total: 42 versus 58 genes; Fig. 2c,e). Within the epidermal compartments, the altered expression levels of 26 genes in NHKs were correlated to NHDF donor age (dark grey), compared to 16 altered gene expression levels that correlated to NHDF body region (light grey; Fig. 2e). Particularly prominent alterations were observed for genes involved in epidermal differentiation (e.g., filaggrin, involucrin, and loricrin, Table S4), epigenetics (e.g., sirtuin-6), and mitochondrial activity (e.g., mitochondrial ribosomal protein L43; Fig. 2f, Table S4). Differences in gene expression were confirmed at the protein level for a selection of genes related to wound healing and epidermal differentiation (Table S4).

### Aged RHS has the hallmarks of stalled wound healing

Next, we investigated the expression of wound healing proteins (Figs 3 and 4). The protein expression of alpha smooth muscle actin (αSMA) and interleukin-6 (IL-6) declined from juvenile to adult to aged RHS (*p* = 0.0002). A reduction in αSMA contributed to rather weak dermal contractility, with an increased surface area seen for the aged RHS (Fig. 4c). Decreased IL-6 levels were in accordance with the thinner dermis of the adult and aged tissues (*p* = 0.0193; Fig. 4a,d). Dermal cell-number decreased markedly from juvenile to aged RHS (*p* = 0.038; Fig. 4b). Alterations in the secretion of pro-matrix metalloproteinase-1 (proMMP-1), HGF, GM-CSF, and IL-6 exceeded even the decreased dermal cell numbers (Fig. 4c,d). Aged NHDFs produced more TNC and laminin-5 than adult and juvenile NHDFs in RHS. Collagen type I alpha 1 gene expression declined (Table S4). Concordantly, two-photon tomography detected only 67% of the collagen I fibrils in aged RHS compared to that in juvenile RHS (Fig. 4c).

Breast skin NHDFs produced less HGF and GM-CSF but more proMMP-1 in adult and aged RHS compared to juvenile RHS (*p* = 0.0137; Fig. 4d). Given the known influence of HGF and GM-CSF on NHK proliferation^20^, the thickness of the viable epidermis decreased by more than 21% (Fig. 4a).

### NHDFs influence epidermal differentiation and *stratum corneum* lipid production

All constructs featured a regular, differentiated, and fully stratified epidermis. However, the epidermal differentiation showed distinct alterations. *Stratum corneum* thickness increased notably in adult and aged RHS compared to juvenile RHS (*p* = 0.0004; Fig. 4a). Moreover, E-cadherin levels declined and filaggrin expression increased in adult and aged RHS (Fig. 3). The total amount of barrier lipids, as well as the lipid/protein ratio, also increased in adult and aged RHS (Fig. S1a). In aged RHS, the ceramide (Cer) levels were markedly increased compared to juvenile RHS (*p* = 0.0256), while the Cer precursors sphingomyelins (SM; *p* = 0.0058) and glucosylceramides (GCer; *p* = 0.0207) were decreased (Fig. 5a). Focusing on the Cer profile, the sphingosine-based Cer classes EOS, NS, and AS were increased in both adult and aged RHS compared to juvenile RHS, but only the increase in Cer NS/NdS was significantly different (*p* = 0.0161; Fig. 5b; for ceramide nomenclature, see Table S5). Additionally, the cholesterol (Chol) and cholesteryl sulfate (CholS) levels were slightly increased in adult and aged RHS, while the free fatty acid (FFA) and phospholipid (PL) levels were slightly decreased (Fig. 5a). Thus, the molar lipid ratio changed from 7:5:1 (FFA:Chol:Cer) in juvenile RHS to 2:3:1 in adult and aged RHS, with the latter being closer to the equimolar lipid ratios found in human skin^21^. We observed a slight enhancement in lipid organization by infrared spectroscopy in adult and aged RHS (Fig. S1b). The altered Cer profile and lower levels of lipid precursors were accompanied by more acidic average surface values in adult and aged RHS (pH 5.1) compared to juvenile RHS (pH 6.0) (Fig. S1c).

### NHDFs impact skin barrier function

Since changes in the lipid profile likely affect barrier function, we studied caffeine and testosterone permeation through juvenile, adult, and aged RHS (Fig. 4c). Caffeine, recommended by the OECD as a probe for hydrophilic drugs, permeated aged RHS less effectively than juvenile or adult RHS. Testosterone, a probe for lipophilic drugs, showed decreased permeation in both adult and aged RHS. Considering the mean apparent permeability coefficient (P_app_), we observed permeation decreases of 30% for caffeine and 40% for testosterone in aged RHS.

Finally, we investigated the RHS penetration of tacrolimus monohydrate (M_r_ 822), a highly lipophilic, anti-inflammatory drug. Despite apparent 4.1- and 6.6-fold increases in the accumulation of tacrolimus in the epidermis of adult and aged RHS relative to juvenile RHS, the total amounts of tacrolimus found in the dermal compartment were equally low in all tested RHS, regardless of the fibroblast origin (Fig. 5d).

## Discussion

The present study investigated an extended range of ageing mechanisms and their differences in juvenile, adult, and aged primary cells instead of using cells in which senescence had been artificially induced or progeroid disease mutations were present^8,22,23^. In particular, our study shows the age-related differences in the dermal expression of wound healing-associated proteins and the impact of body region on epidermal differentiation and drug penetration. We also identified clear alterations to gene expression profiles between primary fibroblasts in 2D monolayer cultures and 3D RHS, emphasizing the need for an organotypic microenvironment *in vitro*.

Since the fibroblasts in the juvenile, adult, and aged RHS differed not only in donor age but also in the body region from which they were isolated, we strictly adhered to the following data analysis outcomes. The effects were termed only age-related when a difference between the models built with fibroblasts from adult and aged donors was observed. We considered differences between RHS built with breast skin fibroblasts and RHS built with foreskin fibroblasts to be localization-related. We considered these differences to be primarily body region-related and only secondarily sex-related, since the experimental setup was free of androgens and oestrogens. The major differences in skin between men and women are caused by hormonal influences, which are regulated systemically *in vivo* and are not represented *in vitro*^24,25^.

The reduced skin thickness, lowered dermal cell number, decrease in collagen I fibrils, and increased proMMP-1 secretion of the aged RHS were well in accordance with aged human skin *in vivo*^26–29^. Nonetheless, the reduced TIMP-1 and collagen I/III expression as well as the increased proMMP-1 expression contributed to the reduced amounts of collagen I fibrils (Fig. 4c). The reduced expression of αSMA along with decreased dermal contraction in aged RHS were in line with impaired wound closing capacity^30^. Furthermore, the increased amounts of TNC observed in aged RHS aligned with chronic non-healing wounds as well as with characteristics of fibrotic diseases such as fibrosis, scleroderma, and liver cirrhosis^31^. The age-dependency of the NHDF proteome fits well with the increased incidence of stalled wound healing in aged patients^5^. While the αSMA and TNC levels varied age-dependently, the GM-CSF and HGF amounts were low in both adult and aged RHS relative to those of juvenile RHS. However, we cannot exclude overlaying effects from donor age and body region. Body region-dependent differences in the gene expression profiles of NHDFs have been previously reported for monolayer cultures^32,33^. Our results indicate that these differences also apply to organotypic cultures. Thus, organotypic models should consider NHDF donor age when testing drugs for delayed wound healing^34^, cardiovascular diseases^35^, and auto-immune^36^ diseases to improve their predictive capacity for the patient group of interest.

The impact of body region and NHDF donor age in our models was not limited to dermal proteins but also influenced epidermal NHKs. Signalling between atopic NHDFs and NHKs has been shown before in three-dimensional skin models^16^, but age-related effects on epidermal-dermal cross-talk have not yet been investigated in conjunction with naturally aged NHDFs. Following full culture in RHS, the donor age of the dermal NHDFs was shown to influence the expression levels of more tested genes in the epidermal NHKs than the body region from which the NHDFs were derived (Fig. 2c). Overall, the expression levels of 26 genes were altered in aged vs. adult RHS, compared to 16 altered gene expression levels in aged and adult vs. juvenile RHS. The gene expression analysis, which covered a wide range of biological processes, showed the marked effect of NHDF donor age on epidermal gene expression. However, at the protein level, the epidermal expression of filaggrin and e-cadherin depended on the body region and less on the donor age of the NHDFs. Nonetheless, this conclusion holds true only for the proteins covered by this study. The increased TGF-β gene expression in aged RHS (Table S4) was in accordance with the lower e-cadherin protein expression and higher fibronectin-1 gene expression that was observed (Fig. 3). This expression pattern is also involved in the epithelial-mesenchymal transition^37^ that occurs during tumourigenesis and might facilitate cancer formation in aged skin.

In addition to GM-CSF and HGF, the IL-6 protein levels decreased from juvenile to adult RHS, and again from adult to aged RHS (Fig. 4d). This finding is in accordance with the changes observed in extracellular matrix-related proteins. *In vivo*, decreased IL-6 levels impair immunity^38^ and foster malignant transformation^39^, both frequent changes in aged skin^5^. In our approach, reduced IL-6 levels, together with low amounts of GM-CSF and HGF, were closely related to increased filaggrin expression (Fig. 3), matching previous studies on filaggrin, GM-CSF, and IL-6 expression^40,41^. Since the filaggrin degradation product *trans*-urocanic acid lowers the pH of the human skin surface by approximately one unit^42^, the marked changes in filaggrin expression outweigh the slightly decreased FFA amounts in adult and aged RHS, and they contribute to the decreased surface pH values. Other contributing factors such as sodium-hydrogen exchanger-1 play a minor role *in vivo*^42^. However, the adult and aged RHS surface pH of 5.1 (Fig. S1c) should also enhance the activity of lipid processing β-glucocerebrosidase and acidic sphingomyelinase^43^; concordantly, increased sphingosine-based Cer levels in the *stratum corneum* were found, and significant decreases in the SM levels were observed (Fig. 5). Overall, the molar ratio of *stratum corneum* lipids in the adult and aged RHS better resembled the equimolar ratio in human skin^21^. Alongside the improved *stratum corneum* lipid profiles, NHDFs slightly improve the *stratum corneum* lipid organization in adult or aged RHS compared to juvenile RHS (Fig. S1b). Moreover, we observed declining caffeine and testosterone permeation through aged RHS (Fig. 5c). This result is in line with the altered lipid profile that was observed as well as with previous skin absorption studies *in vivo*^15,44^.

The morphological changes of adult and aged RHS also affected the skin penetration of tacrolimus following a 5-h exposure to tacrolimus ointment. Tacrolimus amounts in the epidermal compartments of adult and aged RHS greatly exceeded those in juvenile RHS. Tacrolimus penetration into the dermal layer, however, was not affected (Fig. 5d). In total, 0.2% of applied tacrolimus penetrated the dermis, close to the penetration seen in lesional atopic patient skin^45^. The remaining difference can be explained by the well-known differences in the barrier function of skin models and human skin^46^. We hypothesize that this highly lipophilic drug accumulates within the *stratum corneum* and thus pools in greater amounts within the enlarged and lipid-enriched *stratum corneum* of adult and aged RHS (Figs 4a and S1a). Further studies with longer exposure times are needed to elucidate whether this phenomenon also leads to improved tacrolimus uptake in the dermis.

Taken together, our results demonstrate clear differences between monolayer and organotypic culture conditions, suggesting that previous findings in monolayer cultures should be readdressed. These differences are not limited to the skin and are applicable to other tissues as well. In particular, our study shows that age changes the molecular basis of tissue homeostasis. It indicates, for the first time, the impact of NHDF from breast skin and foreskin on the epidermal differentiation and drug penetration of RHS. Thus, we suggest that future preclinical research using RHS can produce results of greater predictive value by using fibroblasts matched to the age and body region of interest. Introducing aged human cells into reconstructed human organs – the skin and beyond – for the study of investigational new drugs should improve the predictability of the results for humans.

## Methods

### Cell culture and human skin reconstruction

Juvenile NHDFs and NHKs were isolated from foreskin (from the medically indicated circumcision of boys younger than 10 years). Adult and aged NHDFs were isolated from breast skin (from plastic surgery; 20- to 30-year-old and 60 to 70-year-old women). The experimental procedures conformed to the principles of the Declaration of Helsinki and were approved by the ethics committees of Charité - Universitätsmedizin Berlin (EA4/091/10, EA1/081/13) and the Medical Faculty of the University of Düsseldorf (#3126). Informed written consent was obtained from all the donors or their parent or legal guardian. RHS was built as previously described^47^. In brief, 8 × 10^5^ NHDFs were embedded into a collagen I matrix (Biochrom, Berlin, Germany) and submerged cultured in construct growth medium (CGM: DMEM/F12 + GlutaMax supplemented with 10% FCS, 1% Pen/Strep, 40 µM adenin HCl monohydrate, 30 µg/L amphotericin B, 0.1 nM choleratoxin, 10 µg/L EGF, 3.5 mg/L hydrocortisone, 4.4 mg/L insulin, 0.5% non-essential amino acids, 4.4 mg/L transferrin, and 2 nM triiodothyronin). After 7 days, 3 × 10^6^ NHKs were seeded onto the dermal equivalents. Airlift was performed the following day, and the models were then cultured in construct differentiation medium (CDM: CGM supplemented with 0.25 mM ascorbic acid and 2 mM calcium chloride) at the air-liquid interface for 14 days.

### Gene expression analysis

Total RNA isolation, cDNA synthesis and quantitative RT-PCR (qPCR) were performed according to established procedures^48^. The primers were designed as described in the Supplementary Information (Table S2) and were synthesised by TIB Molbiol (Berlin, Germany). Fold differences in gene expression were normalised to their respective housekeeping gene, either *YWHAZ*, *GAPDH*, or *RPLP0*. Each housekeeping gene was selected based on its constant expression in all samples for each gene analysis. For PCR array analysis, the epidermis and dermis were separated or the NHDFs were trypsinized, and mRNA was extracted using a NucleoSpin RNA II kit (Macherey-Nagel, Düren, Germany). mRNA was quantified using a NanoQuant Plate and an Infinite200 PRO microplate reader (both Tecan Group Ltd., Männedorf, Switzerland). cDNA synthesis was performed using an RT2 First strand kit (Qiagen, Venlo, Netherlands). Age-related genes were studied using the Human Ageing RT^2^ Profiler PCR Array (Qiagen), and qPCR was performed using a StepOnePlus real-time PCR system (Applied Biosystems, Thermo Fisher Scientific, Waltham, MA, USA) with the RT2 SYSB Green ROX qPCR MasterMix (Qiagen).

### Morphology and immunofluorescence

Each RHS was snap frozen at the end of the culture period, vertically sectioned into 7-µm slices (Leica CM 1510 S; Leica, Wetzlar, Germany), and analysed by haematoxylin-eosin or immunofluorescence staining. Layer thickness and cell counts were analysed with ImageJ software. Pictures were taken with a fluorescence microscope (BZ-8000, Keyence, Neu-Isenburg, Germany) and analysed with BZAnalyzer and ImageJ software. Four individual measurements per construct were performed observer blinded (CH, CZ, and MSK).

### Two-photon tomography

Following the removal of the epidermal layer at the end of the culture period, each dermis preparation was subjected to two-photon tomography (DermaInspect, JenLab, Jena, Germany). Three individual scans per sample were performed 150 µm deep into the dermis with 10-µm increments. The second-harmonic generated light intensity was used to calculate the relative collagen I content compared to that of juvenile RHS. Experimental parameters resemble those of a previously described method^49^.

### Protein quantification

Medium samples were collected at the end of each RHS culture. proMMP-1 and IL-6 were quantified spectrometrically (FLUOstar OPTIMA, BMG Labtech, Ortenberg, Germany) according to the manufacturer’s instructions (proMMP-1, R&D Systems, Minneapolis, MN, USA; IL-6, Affymetrix, Santa Clara, CA, USA). Multiplex analysis was performed for HGF and GM-CSF according to standardized protocols (Bio-Rad, Hercules, CA, USA).

### *Stratum corneum* lipid profile and organization

The *stratum corneum* was isolated from RHS and human skin *ex vivo* and subjected to infrared spectroscopy; subsequently, the lipids were extracted and analysed by high performance thin layer chromatography (HPTLC)^47^. See the Supplementary Information (Table S6) for the standard lipids used and their calibration curve ranges. Non-commercially available Cer EOS and NH standards were synthesised according to previously published methods^50,51^.

### Surface pH

Fluorescence indicator foils using the pH indicator dyes fluorescein isothiocyanate and ruthenium(II)-tris(4,7-diphenyl-1,10-phenanthroline) as a reference (PreSens Precision Sensing, Regensburg, Germany) were gently applied onto the surface of the constructs. Pseudo-colour images produced by VisiSens A2 were transformed into histogram data using VisiSens AnalytiCal 2 and ImageJ^52^.

### Skin permeation

The permeation of radiolabelled [1-methyl^14^C]caffeine (M_r_ 194; log*P* 0.08; PerkinElmer, Waltham, MA, USA) and [1,2,6,7-^3^H]testosterone (M_r_ 293; log*P* 3.32; Amersham, GE Healthcare, Buckinghamshire, UK) was studied in Franz-type diffusion cells as described previously^46^. For the infinite dose approach, we applied solutions of 284.1 µg/cm2 of caffeine or 11.3 µg/cm2 of testosterone (equivalent to 2 mCi/L each) onto the RHS. The permeated amounts of the test compounds were quantified in samples of acceptor medium using a Hidex 300 SL liquid scintillation counter (HIDEX, Turku, Finland).

### Skin penetration

Two hundred micrograms of tacrolimus monohydrate (M_r_ 822; log*P* 3.03) in an ointment (0.1%, Protopic^®^, EurimPharm Arzneimittel, Saaldorf-Surheim, Germany) were applied to RHS^53^. After 5 h, the dermal and epidermal compartments were manually separated and subjected to isotope-dilution LC-MS/MS for tacrolimus quantification as described previously^54^. For a detailed description of the sample preparation, see the Supplementary Information.

### Statistical analysis

Data are presented as the mean ± SD obtained from three to five independent experiments. Due to the explorative data analysis, a level of *p* ≤ 0.05, calculated using non-parametric Kruskal-Wallis tests and subsequent Dunn’s Post hoc tests, was considered to indicate a statistically significant difference.

## Supplementary information

Supplementary Information

## Data Availability

The datasets generated and/or analysed during the current study are available from the corresponding author on reasonable request.
