# Supplementary material for: Fibroblast origin shapes tissue homeostasis, epidermal differentiation, and drug uptake
Source: Sci Rep. 2019 Feb 27;9:2913. doi: 10.1038/s41598-019-39770-6 (PMC6393472; doi:10.1038/s41598-019-39770-6)
Supplement: Supplementary file 1 — Supplementary Information [file 41598_2019_39770_MOESM1_ESM.docx]

**Supplementary Information**

**Fibroblast origin shapes tissue homeostasis, epidermal differentiation, and drug uptake**

Christian Hausmann^1^, Christian Zoschke^1^, Christopher Wolff^1^, Maxim E. Darvin^2^, Michaela Sochorová^3^, Andrej Kováčik^3^, Barbara Wanjiku^1^, Fabian Schumacher^4,5^, Julia Tigges^6^, Burkhard Kleuser^4^, Jürgen Lademann^2^, Ellen Fritsche^6^, Kateřina Vávrová^3^, Nan Ma^7^, and Monika Schäfer-Korting^1,^*

^1^ Institute of Pharmacy (Pharmacology & Toxicology), Freie Universität Berlin, Königin‑Luise‑Str. 2+4, 14195 Berlin, Germany

^2^ Charité - Universitätsmedizin Berlin, Charitéplatz 1, 10117 Berlin, Germany

^3^ Faculty of Pharmacy in Hradec Králové, Charles University, Akademika Heyrovského 1203, 50005 Hradec Králové, Czech Republic

^4^ Institute of Nutritional Science, Department of Nutritional Toxicology, University of Potsdam, Arthur-Scheunert-Allee 114-116, 14558 Nuthetal, Germany

^5^ Department of Molecular Biology, University of Duisburg-Essen, Hufelandstr. 55,
45122 Essen, Germany

^6^ IUF - Leibniz Research Institute for Environmental Medicine, Auf'm Hennekamp 50, 40225 Düsseldorf, Germany

^7^ Institute of Biomaterial Science, Helmholtz‑Zentrum Geesthacht, Kantstraße 55, 14513 Teltow, Germany

*Correspondence

Prof. Dr. Monika Schäfer-Korting

Freie Universität Berlin, Institute of Pharmacy (Pharmacology and Toxicology)

E-Mail: monika.schaefer-korting@fu-berlin.de

**Methods**

**Quantification of tacrolimus in RHS-derived samples using isotope-dilution LC-MS/MS**

All samples were stored at -20 °C until analysis. Skin layer samples (epidermis and dermis) were thawed at room temperature. Then, 15 ceramic beads and 1.5 ml ethanol as extraction solvent containing 1 µM [^13^C_1_,d_4_]tacrolimus (Alsachim, Illkirch Graffenstaden, France) as internal standard were added. Subsequently, skin samples were homogenised for 30 s at high speed using a Bead Ruptor 12 (Omni International, Kennesaw, USA). Homogenisation was repeated once for 60 s. In between both homogenisation steps samples were placed on ice. Afterwards, samples were centrifuged for 10 min at 4.500 *g*. Aliquots of the supernatants were transferred into LC vials and subjected to LC-MS/MS analysis. All analyses were conducted with an Agilent 1260 Infinity LC system coupled to an Agilent 6490 triple quadrupole-mass spectrometer (Agilent Technologies, Waldbronn, Germany) interfaced with an electrospray ion source operating in the positive ion mode (ESI+). Chromatographic conditions and settings of the MS/MS detector for quantification of tacrolimus have been described recently^54^. Briefly, tacrolimus was quantified in relation to its stable isotopically labeled analogue [^13^C_1_,d_4_]tacrolimus using the multiple reaction monitoring (MRM) approach. Ammonium adducts [M + NH_4_]^+^ were selected as precursor ions. The collision-induced loss of two hydroxyl groups and ammonium from the precursor ion, represented by
*m/z* 821.5 → 768.5 for tacrolimus and *m/z* 826.5 → 773.6 for [^13^C_1_,d_4_]tacrolimus (collision energy for both fragmentations: 20 eV), was used for quantification.

**Table S1. Gene list with abbreviations.** Genes are grouped according to their main biological function.

| **Biological Process** | **Gene Name** | **Abbreviation** |
| --- | --- | --- |
| Apoptosis | Caspase 1, apoptosis-related cysteine peptidase | *CASP1* |
|  | Clusterin | *CLU* |
|  | E1A binding protein p300 | *EP300* |
|  | Programmed cell death 6 | *PDCD6* |
|  | Toll interacting protein | *TOLLIP* |
| Cell Cycle | Mitotic Checkpoint Serine/Threonine Kinase B | *BUB1B* |
|  | Cyclin Dependent Kinase Inhibitor 1C | *CDKN1C* |
| Cellular | Cyclin Dependent Kinase Inhibitor 1C | *CDKN1C* |
| Senescence | Von Willebrand Factor A Domain Containing 5A | *VWA5A* |
|  | Werner Syndrome RecQ Like Helicase | *WRN* |
| Cytoskeleton | Alpha 2 smooth muscle Actin | *ACTA2* |
| Regulators | Collagen Type I Alpha 1 Chain | *COL1A1* |
|  | Collagen Type III Alpha 1 Chain | *COL3A1* |
|  | Echinoderm Microtubule Associated Protein Like 1 | *EML1* |
|  | Tenascin C | *TNC* |
| DNA Binding | AT-Rich Interaction Domain 1A | *ARID1A* |
|  | Elongator Acetyltransferase Complex Subunit 3 | *ELP3* |
|  | E1A binding protein p300 | *EP300* |
|  | F-Box And Leucine Rich Repeat Protein 16 | *FBXL16* |
|  | Zinc Finger And BTB Domain Containing 10 | *ZBTB10* |
|  | Zinc Finger RNA Binding Protein | *ZFR* |
|  | Zinc Finger Protein 25 | *ZNF25* |
| Epidermal | Cadherin 1, E-Cadherin | *CDH1* |
| Differentiation | Fibroblast Growth Factor 7, Keratinocyte Growth Factor | *FGF7* |
|  | Filaggrin | *FLG* |
|  | Involucrin | *IVL* |
|  | Loricrin | *LOR* |
|  | Transforming Growth Factor Beta 1 | *TGFB1* |

**Table S1. Continued**

| **Biological Process** | **Gene Name** | **Abbreviation** |
| --- | --- | --- |
| Epigenetic | AT-Rich Interaction Domain 1A | *ARID1A* |
| Alterations | Sirtuin 1 | *SIRT1* |
|  | Sirtuin 3 | *SIRT3* |
|  | Sirtuin 6 | *SIRT6* |
| Extracellular | Alpha 2 smooth muscle Actin | *ACTA2* |
| Matrix | Collagen Type I Alpha 1 Chain | *COL1A1* |
|  | Collagen Type III Alpha 1 Chain | *COL3A1* |
|  | Fibronectin 1 | *FN1* |
|  | Laminin Subunit Beta 3 | *LAMB3* |
| Genomic | Mitotic Checkpoint Serine/Threonine Kinase B | *BUB1B* |
| Instability | Mitochondrial Ribosomal Protein L43 | *MRPL43* |
|  | RNA Polymerase Mitochondrial | *POLRMT* |
|  | Mitochondrial Transcription Factor A | *TFAM* |
|  | Mitochondrial Transcription Factor B1 | *TFB1M* |
|  | Mitochondrial Transcription Factor B2 | *TFB2M* |
|  | Zinc Metallopeptidase STE24 | *ZMPSTE24* |
| Housekeeping | Actin Beta | *ACTB* |
| Genes | Beta-2-Microglobulin | *B2M* |
|  | Glyceraldehyde-3-Phosphate Dehydrogenase | *GAPDH* |
|  | Hypoxanthine Phosphoribosyltransferase 1 | *HPRT1* |
|  | Ribosomal Protein Lateral Stalk Subunit P0 | *RPLP0* |
|  | Tyrosine 3-Monooxygenase/Tryptophan 5-Monooxygenase Activation Protein Zeta | *YWHAZ* |
| Inflammatory | Angel Homolog 2 | *ANGEL2* |
| Response | Annexin A3 | *ANXA3* |
|  | Annexin A5 | *ANXA5* |
|  | Complement C1q A Chain | *C1QA* |
|  | Complement C1q B Chain | *C1QB* |

**Table S1. Continued**

| **Biological Process** | **Gene Name** | **Abbreviation** |
| --- | --- | --- |
| Inflammatory | Complement C1q C Chain | *C1QC* |
| Response | Complement C1s | *C1S* |
|  | Complement C3 | *C3* |
|  | Complement C3a Receptor 1 | *C3AR1* |
|  | Complement C5a Receptor 1 | *C5AR1* |
|  | C-C Motif Chemokine Receptor 1 | *CCR1* |
|  | CD14 Molecule | *CD14* |
|  | CD163 Molecule | *CD163* |
|  | Complement Factor H | *CFH* |
|  | C-X3-C Motif Chemokine Ligand 1 | *CX3CL1* |
|  | C-X-C Motif Chemokine Ligand 16 | *CXCL16* |
|  | Fc Fragment Of IgE Receptor Ig | *FCER1G* |
|  | Fc Fragment Of IgG Binding Protein | *FCGBP* |
|  | Fc Fragment Of IgG Receptor Ia | *FCGR1A* |
|  | Fc Fragment Of IgG Receptor IIa | *FCGR2A* |
|  | Fc Fragment Of IgG Receptor IIIb | *FCGR3B* |
|  | Glial Fibrillary Acidic Protein | *GFAP* |
|  | Interleukin 6 | *IL6* |
|  | Interleukin 8 | *IL8* |
|  | Lactotransferrin | *LTF* |
|  | Lysozyme | *LYZ* |
|  | Myelin Basic Protein | *MBP* |
|  | Pannexin 1 | *PANX1* |
|  | S100 Calcium Binding Protein A8 | *S100A8* |
|  | S100 Calcium Binding Protein A9 | *S100A9* |
|  | Toll Like Receptor 2 | *TLR2* |
|  | Toll Like Receptor 4 | *TLR4* |
|  | Transmembrane Protein 135 | *TMEM135* |
|  | Transmembrane Protein 33 | *TMEM33* |
|  | Tumor Necrosis Factor | *TNF* |
|  | Toll Interacting Protein | *TOLLIP* |

**Table S1. Continued**

| **Biological Process** | **Gene Name** | **Abbreviation** |
| --- | --- | --- |
| Laminopathies | Lamin A/C | *LMNA* |
|  | Lamin B1 | *LMNB1* |
|  | Lamin B2 | *LMNB2* |
|  | Zinc Metallopeptidase STE24 | *ZMPSTE24* |
| Mitochondrial | Mitochondrial Ribosomal Protein L43 | *MRPL43* |
| Dysfunction | NADH:Ubiquinone Oxidoreductase Subunit B11 | *NDUFB11* |
|  | Mitochondrial RNA Polymerase | *POLRMT* |
|  | Sirtuin 1 | *SIRT1* |
|  | Sirtuin 3 | *SIRT3* |
|  | Sirtuin 6 | *SIRT6* |
|  | Mitochondrial Transcription Factor A | *TFAM* |
|  | Mitochondrial Transcription Factor B1 | *TFB1M* |
|  | Mitochondrial Transcription Factor B2 | *TFB2M* |
| Neurodegeneration & | Calbindin 1 | *CALB1* |
| Synaptic | Glial Fibrillary Acidic Protein | *GFAP* |
| Transmission | Myelin Basic Protein | *MBP* |
|  | Sodium Voltage-Gated Channel Beta Subunit 2 | *SCN2B* |
|  | Synaptosome Associated Protein 23 | *SNAP23* |
| Oxidative | E1A binding protein p300 | *EP300* |
| Stress | Glutathione S-Transferase Alpha 1 | *GSTA1* |
| Proteostasis | ADP Ribosylation Factor Like GTPase 6 Interacting Protein 6 | *ARL6IP6* |
|  | Mitotic Checkpoint Serine/Threonine Kinase B | *BUB1B* |
|  | Forkhead Box O1 | *FOXO1* |
|  | Heat Shock Transcription Factor 1 | *HSF1* |
|  | Janus Kinase And Microtubule Interacting Protein 3 | *JAKMIP3* |
|  | Ring Finger Protein 144B | *RNF144B* |
|  | Sirtuin 1 | *SIRT1* |
|  | Thioredoxin Interacting Protein | *TXNIP* |
|  | Vacuolar Protein Sorting 13 Homolog C | *VPS13C* |

**Table S1. Continued**

| **Biological Process** | **Gene Name** | **Abbreviation** |
| --- | --- | --- |
| RNA Binding | ELAV Like RNA Binding Protein 1 | *ELAVL1* |
|  | LSM5 Homolog, U6 Small Nuclear RNA And MRNA Degradation Associated | *LSM5* |
|  | Zinc Finger RNA Binding Protein | *ZFR* |
| Telomere | Protection Of Telomeres 1 | *POT1* |
| Attrition | RAP1A, Member Of RAS Oncogene Family | *RAP1A* |
|  | Telomeric Repeat Binding Factor 1 | *TERF1* |
|  | Telomeric Repeat Binding Factor 2 | *TERF2* |
|  | TERF1 Interacting Nuclear Factor 2 | *TINF2* |
|  | Tripeptidyl Peptidase 1 | *TPP1* |
| Transcriptional | AT-Rich Interaction Domain 1A | *ARID1A* |
| Regulation | E1A binding protein p300 | *EP300* |
|  | Forkhead Box O1 | *FOXO1* |
|  | Heat Shock Transcription Factor 1 | *HSF1* |
|  | PHD Finger Protein 3 | *PHF3* |
|  | SMAD Family Member 2 | *SMAD2* |
| Wound | Alpha 2 smooth muscle Actin | *ACTA2* |
| Healing | Colony Stimulating Factor 2, Granulocyte-Macrophage Colony Stimulating Factor | *CSF2* |
|  | Fibronectin 1 | *FN1* |
|  | Hepatocyte Growth Factor | *HGF* |
|  | Interleukin 6 | *IL6* |
|  | Interleukin 8 | *IL8* |
|  | Matrix Metalloproteinase 1 | *MMP1* |
|  | Matrix Metalloproteinase 3 | *MMP3* |
|  | Transforming Growth Factor Beta 1 | *TGFB1* |
|  | Tenascin C | *TNC* |
|  | Vascular Endothelial Growth Factor C | *VEGFC* |

**Table S2. Primer sequences for PCR studies.** All other genes were studied using the Human Aging RT^2^ Profiler PCR Array (SaBiosciences, Qiagen). For primer sequences, the reader is referred to the manufacturer.

| **Gene Name** | **Used primer sequence** | |
| --- | --- | --- |
|  | **Forward** | **Reverse** |
| *ACTA2* | TGGGCTCTGTAAGGCCGGCT | TCACCCCCTGATGTCTGGGACG |
| *CDH1* | GAAGGTGACAGAGCCTCTGGAT | GATCGGTTACCGTGATCAAAATC |
| *CSF2* | TCTCAGAAATGTTTGACCTCCA | GCCCTTGAGCTTGGTGAG |
| *FGF7* | AAGTTGCACCAGGCAGACA | CGCTGTTTGCTATTTGACTTTTGT |
| *FLG* | TGAAGCCTATGACACCACTGA | TCCCCTACGCTTTCTTGTCCT |
| *FN1* | CCCAATTGAGTGCTTCATGCC | GCTGGGTCTGCTAACATCACT |
| *HGF* | TCACGAGCATGACATGACTCC | AGCTTACTTGCATCTGGTTCC |
| *IL6* | CACAGACAGCCACTCACCTC | TTTTCTGCCAGTGCCTCTTT |
| *IL8* | CAAGAGCCAGGAAGAAACCA | GTCCACTCTCAATCACTCTCAG |
| *IVL* | TCCTCCAGTCAATACCCATCAG | CAGCAGTCATGTGCTTTTCCT |
| *LAMB3* | GCCCATGAATGCCAAAGGTG | CAAACACAGCGGGGTCAAAG |
| *LOR* | TCATGATGCTACCCGAGGTTTG | CAGAACTAGATGCAGCCGGAGA |
| *MMP1* | GGGAGATCATCGGGACAACTC | GGGCCTGGTTGAAAAGCAT |
| *MMP3* | TGGACAAAGGATACAACAGGGAC | AGCTTCAGTGTTGGCTGAGT |
| *RPLP0* | GCTGCTGCCCGTGCTGGTG | TGGTGCCCCTGGAGATTTTAGTGG |
| *TGFB* | CACCCGCGTGCTAATGG | ATGCTGTGTGTACTCTGCTTGAACT |
| *TNC* | TAGTGGTCAAGTGGGAGGGG | AGCTTTTCCCAAGTGTGTTCA |
| *TNFA* | CCCAGGGACCTCTCTCTAATCA | GCTACAGGCTTGTCACTCGG |
| *VEGFC* | TCAGGCAGCGAACAAGACCT | TTCCTGAGCCAGGCATCTG |
| *YWHAZ* | AGACGGAAGGTGCTGAGAAA | GAAGCATTGGGGATCAAGAA |

**Table S3. Gene expression of NHDF in monolayer culture and RHS.** The rows correspond to the respective comparisons in Venn diagram (Fig. 2A) and consider fold changes in gene expression > |1.3| and Ct values ≤ 35.

| **NHDF: Monolayer vs RHS** | **Upregulation** | **Downregulation** |
| --- | --- | --- |
| juvenile adult aged | *ANXA3, C3, C3AR1, C5AR1, CFH, CXCL16, FOXO1, PANX1, TLR2* | *ANGEL2, ANXA5, ARID1A, BUB1B, COL1A1, ELAVL1, LMNA, LMNB1, LMNB2, MBP, MRPL43, NDUFB11, PDCD6, POT1, RAP1A, RNF144B, SIRT3, SNAP23, TERF1, TFB2M, TLR4, TMEM135, TMEM33, ZFR, ZMPSTE24* |
| adult aged | *CASP1, CD14, COL3A1, EML1, EP300, PHF3, SIRT1, VPS13C, WRN, ZBTB10, ZNF25* | *CLU, TXNIP* |
| aged | *--* | *FCER1G, VWA5A* |
| juvenile aged | *FBXL16* | *C1S, TOLLIP* |
| juvenile | *CLU, LSM5* | *CASP1, CD14* |
| juvenile adult | *CDKN1C* | *--* |
| adult | *SMAD2, TERF2, TFAM, TPP1* | *FBXL16, TFB1M* |

**Table S4. Gene expression of NHDF and keratinocytes in RHS.** The rows correspond to the respective comparisons in Venn diagrams (Fig. 2C, E) and consider fold changes in gene expression > |1.3| and Ct values ≤ 35. Changes due to donor age (dark grey) and body area of the NHDF (light grey).

| **NHDF in RHS** | **Upregulation** | **Downregulation** |
| --- | --- | --- |
| adult vs. juvenile aged vs. juvenile aged vs. adult | *CD163, FN1, MMP1, TNC* | *C3* |
| aged vs. juvenile aged vs. adult | *FGF7, TGFB* | *CFH, COL1A1, VEGFC* |
| aged vs. adult | *C3AR1, CSF2, IL6, WRN* | *IL8, S100A8, S100A9, TNF* |
| adult vs. juvenile aged vs. adult | *--* | *--* |
| adult vs. juvenile | *LSM5, TOLLIP, VEGFC* | *ACTA2, ANXA5, ARID1A, ARL6IP6, CDKN1C, IL6* |
| adult vs. juvenile aged vs. juvenile | *ANXA3, C5AR1, CASP1, CD14, CXCL16, FOXO1, HGF, IL8, MBP, MMP3, S100A8, S100A9, TNF, TLR2, TLR4, TXNIP, VWA5A* | *ANGEL2, C1S, C3AR1, CLU, COL3A1, CSF2, CX3CL1, EML1, EP300, FCGBP, LMNA, LMNB1, PANX1, PHF3, SIRT1, TFAM, TFB2M, WRN, ZBTB10, ZFR* |
| aged vs. juvenile | *FBXL16* | *NDUFB11, TERF1* |
| **NHK in RHS** | ***Upregulation*** | ***Downregulation*** |
| adult vs. juvenile aged vs. juvenile aged vs. adult | *FLG, TXNIP* | *FCGR1A* |
| aged vs. juvenile  aged vs. adult | *C3, CD14, S1PR1* | *CDH1, FBXL16, HSF1, VEGFC* |
| aged vs. adult | *C1S, CDKN1C, TLR2* | *ANGEL2, COL1A1, COL3A1, EML1, FCER1G, IVL, JAKMIP3, LMNB2, LOR, MRPL43, POLRMT, SIRT3, SIRT6, ZNF25* |
| adult vs. juvenile  aged vs. adult | *--* | *--* |

**Table S4. Continued**

| **NHK in RHS** | **Upregulation** | **Downregulation** |
| --- | --- | --- |
| adult vs. juvenile | *CDH1, ELP3, EML1, FCER1G, IVL, JAKMIP3, LSM5, POT1, RNF144B, SIRT3, SIRT6, TINF2, VEGFC, ZNF25* | *ANXA3, C3, CDKN1C, TFAM, TLR2, TMEM135* |
| adult vs. juvenile aged vs. juvenile | *BUB1B, CASP1, CFH, COL1A1, COL3A1, LOR, ZMPSTE24* | *CLU, VWA5A* |
| aged vs. juvenile | *S100A8* | *EP300* |

**Table S5. Ceramide nomenclature.** (Reprinted from Zoschke et al. 2016^47^ with permission from Elsevier).

**

**

**Table S6. Calibration curve ranges of lipid standards used for HPTLC analysis.**

| **Lipid standard** | | **Calibration curve range [µg]** | | |
| --- | --- | --- | --- | --- |
| FFA | Lignoceric acid | 0.5 | - | 12.5 |
| Chol | Cholesterol | 0.5 | - | 12.5 |
| Cer | Ceramide EOS | 0.05 | - | 1.25 |
|  | Ceramide NS | 0.2 | - | 5 |
|  | Ceramide NP | 0.4 | - | 9.5 |
|  | Ceramide AS | 0.1 | - | 2.5 |
|  | Ceramide NH | 0.15 | - | 4 |
|  | Ceramide AP | 0.1 | - | 2.5 |
| CholS | Cholesterol sulfate | 0.105 | - | 2.474 |
| SM | Sphingomyelin | 0.211 | - | 4.947 |
| GCer | Glucosylceramide | 0.263 | - | 6.184 |
| PL | Phospholipid | 0.421 | - | 9.895 |

**Figure S1**


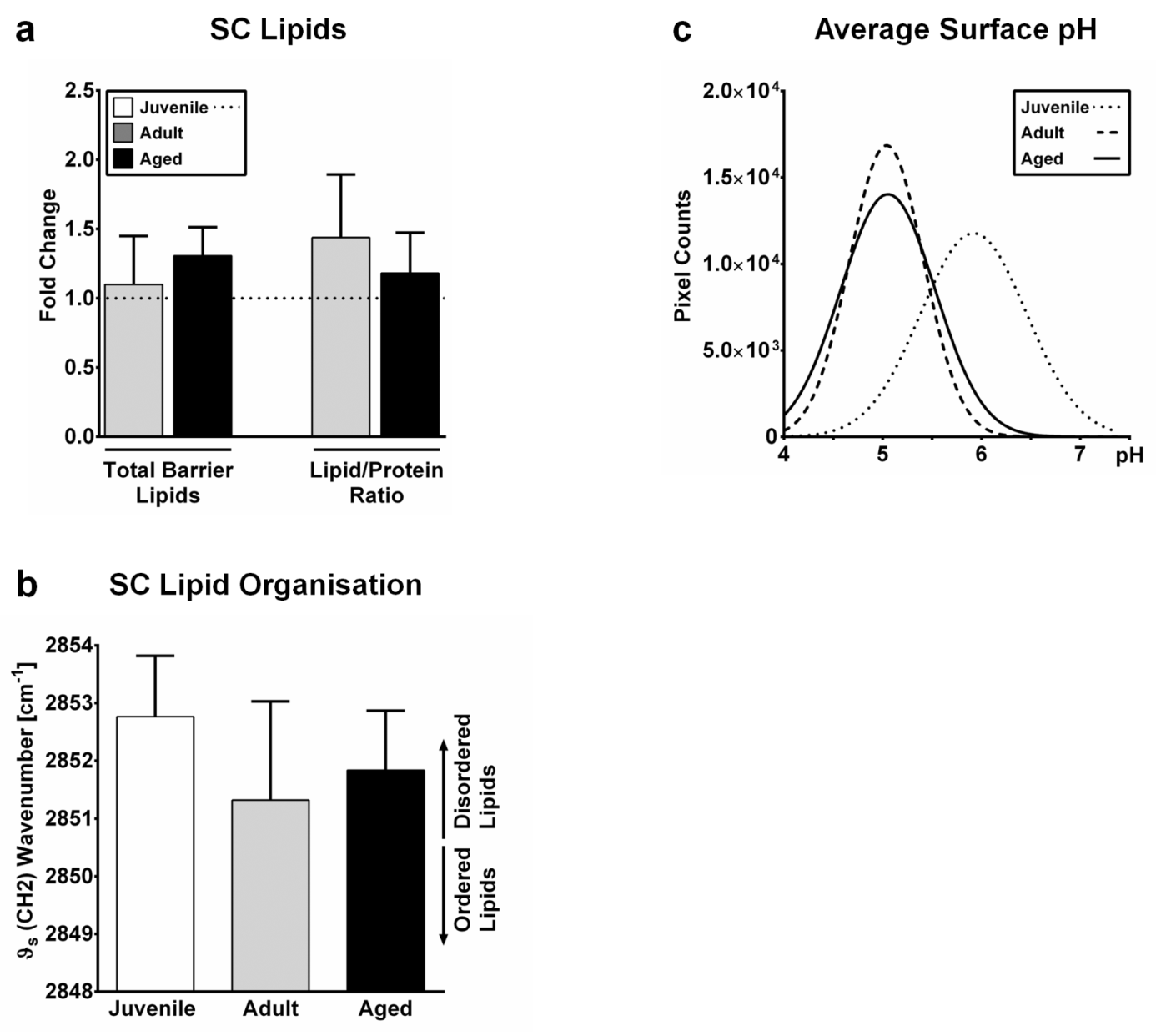


**Fig. S1.** Impact of normal human dermal fibroblast donor age and body region on *stratum corneum* (SC) lipids. (**A**) Amount of total SC lipids and lipid/protein compared to juvenile RHS; dotted line, fold changes in comparison to juvenile RHS. (**B**) SC lipid organisation, measured as methylene stretching vibration in RHS. (**C**) Average surface pH. Graphs depict data of three batches and are presented as the mean ± SD.
